# Letter to the Editor concerning Takeda M, Koga H, Lane GJ, Tanaka N, Nagakawa Y, Okazaki T, Urao M,Yamataka A. Historical aspects of anatomic landmarks during pull-through for hirschsprung disease: focusing on resection levels of the aganglionic rectum and rectal cuff issues. Pediatr Surg Int. 2025 Nov 17;42(1):14

**DOI:** 10.1007/s00383-026-06366-x

**Published:** 2026-03-04

**Authors:** Miriam Wilms, Mazeena Mohideen, Madelaine Neumayr, Annette Lemli

**Affiliations:** 1 Patient Organization for People with Anorectal Malformations and Hirschsprung Disease (SoMA e.V.), Munich, Germany; 2 Faculty of Health/School of Medicine, Witten/Herdecke University, Witten, Germany; 3 Patient Organization for People with Anorectal Malformations and Hirschsprung Disease (SoMA Austria), Vienna, Austria

Dear Editor,

We would like to express our gratitude to the authors of the article “Historical aspects of anatomic landmarks during pull-through forhirschsprung disease: focusing on resection levels of the aganglionicrectum and rectal cuff issues” for their comprehensive historical analysis of how the understanding and surgical management of Hirschsprung disease have evolved over time. As Austrian and German patient organizations representing individuals of all ages affected by Hirschsprung disease, we witness the long-term outcomes across different “generations” of patients and the corrective procedures they have undergone.

In recent years, we have become increasingly alarmed about the growing number of members with Hirschsprung disease suffering from fecal incontinence, particularly within the younger generation treated with transanal pull-through procedures. A careful examination of the technique specific challenges, especially with regard to preservation of the anal canal and the continence mechanism, is therefore of utmost importance from a patient perspective.

Takeda et al. demonstrate that resection margins in pull-through surgery for Hirschsprung disease have been defined inconsistently for decades, often without sufficient consideration of the anatomy and physiology of the anal canal. They further highlight that the high rate of postoperative fecal incontinence, particularly following the introduction of the transanal approach, has prompted a necessary re-evaluation of overly distal resection margins. In addition, the authors draw attention to the inconsistent and occasionally contradictory terminology used to describe the relevant anatomical structures.

The statement by Takeda et al. that “Swenson’s results could not be replicated by less experienced surgeons at the time” underscores an important distinction between outcomes achieved under ideal conditions and those observed in routine clinical practice. Real-world results depend not only on the theoretical advantages of a technique but also on its complexity, the quality of surgical training, and the manner in which it is introduced into broader clinical use.

While the critical appraisal of surgical techniques is rightly the domain of scientific research, we believe it is equally important to examine how these techniques are implemented within contemporary care structures across different countries. Ultimately, real-world outcomes define the daily challenges faced by affected individuals, and fecal incontinence remains among the most devastating complications.

In support of the authors’ recommendation that readers “refresh their understanding of the terminology used to describe surgical landmarks in the anorectum”, we are convinced that consistent terminology, particularly the recognition of the anorectal line as the key landmark at the oral end of the anal columns for defining the distal resection margin, may contribute to improved outcomes for future generations of patients with Hirschsprung disease.

We hope that this article will stimulate an important discussion on how this essential aspect of patient safety can be ensured for all individuals undergoing corrective surgery for Hirschsprung disease.

Further research may help clarify whether synoptic operative reporting or standardized pathological assessment of the distal resection margin could improve surgical precision, how visualization of the anorectal line might be optimized intraoperatively, and how fecal continence can best be preserved in cases requiring redo surgery with an anastomosis close to this landmark.

As emphasized by Carl Sagan, “You have to know the past to understand the present”, this article represents an important step toward that understanding.

Sincerely yours,

Miriam Wilms, Mazeena Mohideen, Madelaine Neumayr, Annette Lemli

## Data Availability

No datasets were generated or analysed during the current study.

